# CRISPR/Cas9-mediated LINC00511 knockout strategies, increased apoptosis of breast cancer cells via suppressing antiapoptotic genes

**DOI:** 10.1186/s12575-022-00171-1

**Published:** 2022-07-05

**Authors:** Narjes Azadbakht, Abbas Doosti, Mohammad-Saeid Jami

**Affiliations:** 1 Department of Biology, Faculty of Basic Sciences, Shahrekord Branch, Islamic Azad University, Shahrekord, Iran; 2 Biotechnology Research Center, Shahrekord Branch, Islamic Azad University, Shahrekord, Iran; 3 Cellular and Molecular Research Center, Basic Health Sciences Institute, Shahrekord University of Medical Sciences, Shahrekord, Iran

**Keywords:** Breast cancer, LINC00511, CRISPR/Cas9, Knockout

## Abstract

**Background:**

The growing detection of long noncoding RNAs (lncRNAs) required the application of functional approaches in order to provide absolutely precise, conducive, and reliable processed information along with effective consequences. We utilized genetic knockout (KO) techniques to ablate the Long Intergenic Noncoding RNA 00,511 gene in several humans who suffered from breast cancer cells and at the end we analyzed and examined the results.

**Results:**

The predictive relevance of LINC00511 expression pattern was measured by using a pooled hazard ratio (HR) with a 95% confidence interval (CI). The link among LINC00511 expression profiles and cancer metastasis was measured by using a pooled odds ratio (OR) with a 95% confidence interval. This meta- analysis was composed of fifteen studies which contained a total of 1040 tumor patients. We used three distinct CRISPR/Cas9-mediated knockdown techniques to prevent the LINC00511 lncRNA from being transcribed. RT-PCR was used to measure lncRNA and RNA expression. We used CCK-8, colony formation tests, and the invasion transwell test to measure cell proliferation and invasion. The stemness was measured by using a sphere-formation test. To validate molecular attachment, luciferase reporter assays were performed. The functional impacts of LINC00511 gene deletion in knockdown breast cancer cell lines were confirmed by using RT-qPCR, MTT, and a colony formation test. This meta-analysis was composed of 15 trials which contained a total of 1040 malignant tumors. Greater LINC00511 expression was ascribed to a lower overall survival (HR = 1.93, 95% CI 1.49–2.49, < *P* 0.001) and to an increased proportion of lymph node metastasis (OR = 3.07, 95% CI 2.23–4.23, *P* < 0.001) in the meta‐analysis. It was found that the role of LINC00511 was overexpressed in breast cancer samples, and this overexpression was ascribed to a poor prognosis. The gain and loss-of-function tests demonstrated findings such as LINC00511 increased breast cancer cell proliferation, sphere-forming ability, and tumor growth. Additionally, the transcription factor E2F1 binds to the Nanog gene's promoter site to induce transcription. P57, P21, Prkca, MDM4, Map2k6, and FADD gene expression in the treatment group (LINC00511 deletion) was significantly higher than in the control group (*P* < 0.01). In addition, knockout cells had lower expression of BCL2 and surviving genes than control cells *P* < 0.001). In each of the two target alleles, the du-HITI approach introduced a reporter and a transcription termination signal. This strategy's donor vector preparation was significantly easier than "CRISPR HDR," and cell selection was likewise much easier than "CRISPR excision." Furthermore, when this approach was used in the initial transfection attempt, single-cell knockouts for both alleles were generated.

**Conclusions:**

The methods employed and described in this work could be extended to the production of LINC00511 knockout cell lines and, in theory, to the deletion of other lncRNAs to study their function.

**Supplementary Information:**

The online version contains supplementary material available at 10.1186/s12575-022-00171-1.

## Background

Breast cancer was among the most frequent malignancies in women all over the world, and it was the main cause of the disease death among women [1, 2]. Surgery, chemotherapy, and/or radiation therapy were the most common treatments for breast cancer, and they improved the therapeutic benefits significantly [3]. Based on various studies, Breast cancer persistence and regeneration were linked to stem cell features [4]. Cancer stem cells (CSCs) or tumor-initiating cells could be deemed as a subtype of cancerous cells which were present in the malignancy. CSCs had the ability to self-renew, differentiate in multiple directions, proliferate indefinitely, and repair tumors. They were linked to tumor growth, division, invasion, and metastasis, as well as radio-chemotherapy tolerance and recurrent [5]. Breast cancer stem cells (BCSCs) were proved to have an important role in the reproduction and self-renewal capability of tumor cells [6].

Long non-coding RNAs (lncRNAs) could be utilized in the control of both coding and non-coding RNA synthesis and post-transcriptional activities. it was shown that lncRNAs could perform miscellaneous functions such as guiding chromatin regulators to specific genomic regions, sequestration transposable elements, allosteric regulation of transcriptional molecules, altering cytoplasmic domains, modulating translation, modulating mRNA viability, and acting as competitive internal RNAs [7–10]. The significance of lncRNAs in biological processes such as genomic methylation, pharmacological adjustment, pluripotency, and differentiation determination [11, 12] was discovered by different studies to emphasize their role in developmental stages and their abnormal performance in various disorders. LncRNAs proved to be biologically important as a result of these discoveries. Functional studies also accentuated the significance of lncRNAs [13]. A variety of investigations planned and carried out to examine the role of lncRNAs. There were three types of studies. RNA interference (RNAi) and antisense nucleotide sequences were two types of antisense oligonucleotides that worked to destroy synthesized lncRNA. The second group included CRISPR-mediated interference (CRISPRi) and activation (CRISPRa), which engage the gene's regulatory regions to regulate its expression level [14]. Configurable nucleases, such as zinc-finger nucleases (ZFNs), transcription activator-like effector nucleases (TALENs), and CRISPR/Cas9 (CRISPR-associated protein-9 nuclease), were used to ablate genes in the third group. For the correct combination of a practical investigation for a particular transcription, many factors were suggested [14, 15]. These parameters were mostly concerned with the targeted lncRNA gene's region and position concerning other genes, as well as the placement of its regulatory areas [15] and lncRNA's sub-cellular placement. Knocking down an lncRNA to explore its action had at least two drawbacks: (1) partial transcript reduction owing to the structure of the technique and the nuclear placement of lncRNA, and (2) probable off-target consequences [16, 17]. When the goal gene's expression was controlled in a sophisticated way, CRISPRi and CRISPRa procedures might fail to block or stimulate lncRNA transcription [18]. CRISPR/Cas9 dual allele homology-independent targeted integration (CRISPR du-HITI) was a technique in which a targeting segment replaced the genetic area across two Cas9 double-strand breaks (DSBs). By aiming at two different bases for genic manipulation, we can place two genotypes in one identical chromosomic area. In other words, in one identical area, we did two chromosomic manipulation to place two genotypes.

Several lnc.RNAs, including NEAT1, LINC00473, and LINC01133 emerged to have a role in cancer carcinogenesis [19]. According to Sun et al., NEAT1 acted as a carcinogen in cancer by functioning as a competitive endogenous RNA (ceRNA) for miR-377-3p, resulting in the de-suppression of E2F3, an endogenous goal of miR-377-3p and a major oncogene in cancer development [20]. LINC00473 was a nucleus lncRNA which was strongly expressed in cancer, and increased LINC00473 transcription could be linked to a bad prognosis [19]. However, the precise molecular processes of lncRNAs in breast cancer have yet to be fully elucidated. Long intergenic noncoding RNA 00,511 (LINC00511) was discovered as a recently discovered lncRNA that is elevated in human breast cancer and may act as an oncogene [21]. Despite these findings, the biological functions of LINC00511 in breast carcinogenesis and the biochemical processes that underpinned them were still unknown. The oncogene LINC00511 (LINC00511) influenced tumor growth, migration, and poor outcome. The histone methyltransferase EZH2 interacted with LINC00511 and defined the histone alteration profile on p57 [22]. In cancer and pancreatic ductal adenocarcinoma, it also acted as an oncogene [19, 21, 22]. We screened for differentially regulated lncRNAs for breast cancer on a broad scale before the inception of our study. We chose the LINC00511 as the objective after screening and evaluation. LINC00511 was found to be a carcinogenic RNA in the development of breast cancer tumors in the present study. We used a CRISPR/Cas9 variant in the current study. In this technique, the targeted segment replaced the genomic region positioned between two Cas9-induced double-strand breaks (DSBs) in each allele. CRISPR du-HITI has two functioning arms which can place two alleles in one chromosomic area simultaneously.

Knockdown techniques were effectively applied and utilized, and experimental cell lines missing the LINC00511 lncRNA were created.

## Results

### Results of study selection and characteristics

Online databases resulted in a total of 314 possibly related articles. To find articles that were qualified, we first eliminated 128 duplicates. We eliminated 95 papers after reviewing the titles and abstracts of conferences papers, review articles, and studies which were unrelated to our study topic. The full text of 91 articles was then studied. Due to inadequate data and TCGA data, 76 studies were excluded during this stage. Finally, this meta-analysis included a total of 15 papers that served our purpose right. Figure S1 showed a flowchart that summarized the study recruitment process. Between 2015 and 2021, all of the research studies were checked. The sample size of the study that was eligible varied from 30 to 150 people. MDA-MB-468 (*n* = 2), MDA-MB-231 (*n* = 3), MDA-MB-453 (*n* = 3), and MCF-7 (*n* = 7) were among the cancer cell lines studied. RT-qPCR was used to identify LINC00511 target gene expression in all of the investigations that were qualified. Based on NOS standards, all of the included articles received at least six points, indicating that they were of reasonably high quality and hence suitable for this meta-analysis.

### Correlation between LINC00511 overexpression and survival outcomes

**Fig. 1:**
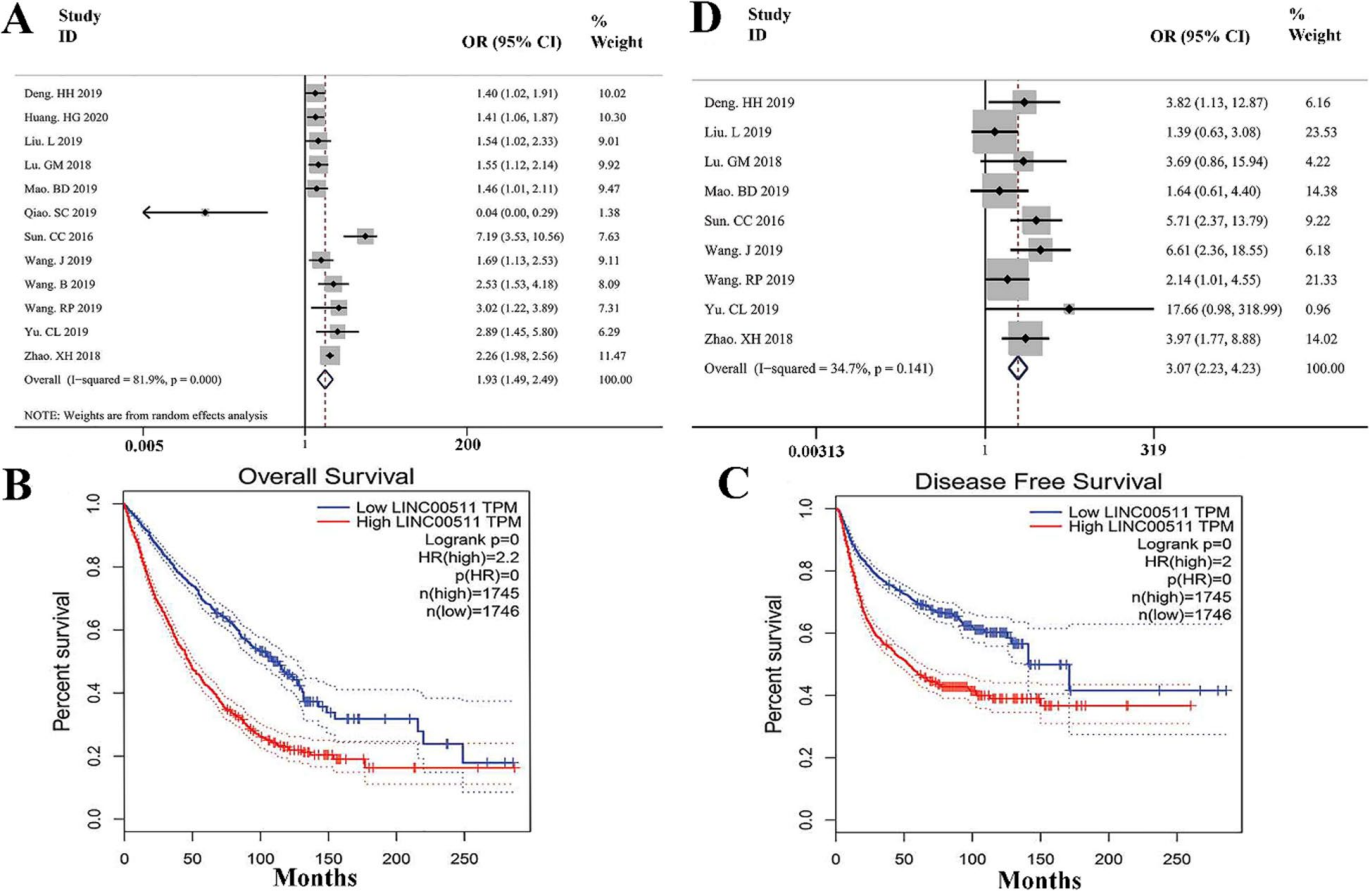
**A** forest plot showing the correlation between LINC00511 overexpression and general survival in patients with breast tumors. **B** Kaplan–Meier survival evaluation of LINC00511 production for overall survival and, **C** disease-free survival in patients with breast cancers; **D** The relationship between LINC00511 overexpression and lymph node metastases is depicted as a forest plot

We selected 15 studies with a total of 1040 patients with metastatic tumors in our meta-analysis to analyze the link between LINC00511 overexpression and OS. The random-effect model was used to compute the pooled HR and 95 percent CI for OS because of the evident heterogeneity across these trials (I5 = 81.9%, *p *< 0.01). Higher LINC00511 production was associated with a worse chance of survival (1.93, 95% CI 1.49–2.49, *P* < 0.001) (Fig. 1 A). We were unable to perform a meta-analysis of the prognostic value of LINC00511 production for these survival outcomes as only 2 studies documented the connection between LINC00511 production and progression-free survival, and none of the studies were pertinent to DFS. To verify the findings of our meta-analysis and measure the prognostic value of LINC00511 transcription for other survival rates, survival plots were created by using GEPIA via combining LINC00511 expression data with malignancy survival data from the entire TCGA dataset, in which 3491 patients were divided into maximum and minimum expression groups based on LINC00511 production. The findings evinced that increased LINC00511 expression was associated with worse OS and DFS (Fig. 1B, C), confirming the findings of this meta-analysis.

### The relationship between LINC00511 expression and lymph node metastases was studied in a meta-analysis

This meta-analysis examined 8 papers contained 833 tumor patients to see whether there was a link between LINC00511 production and lymph node metastases. We used the fixed-effect model to determine the pooled OR and 95 percent CI for lymph node metastasis since there was no substantial variance across these investigations (I5 = 34.7 percent, *p* = 0.141). Increasing LINC00511 expression was shown to be associated with a higher risk of lymph node metastasis (OR = 3.07, 95 percent CI 2.23–4.23, *P* < 0.001). (Fig. 1D). It was impossible to perform a study such as a meta-analysis to explore the connection between LINC00511 expression and distant metastasis due to the limited sample group since there existed only two studies which showed the connection between LINC00511 production and metastatic disease so far.

Based on bioinformatics findings, the apoptosis-related genes in breast cancer, mutation frequency and copy number change (CNV) is different.

In Fig S3 A, clueGo was used to map the apoptotic gene pathways that were associated with LINC00511. Based on the QIAGEN database and the LINC00511 gene, all apoptotic genes were discovered (Fig S3 B). Afterwards, LINC00511's L network was presented. All genes related to LINC00511 mice were retrieved from the L database and shown in Fig S3 C. In both the L network and the expression association with LINC00511, the prkca gene was the sole gene which was present. The network of RNA internal competition for LINC00511 was shown. Figure S3 D depicted the RNA internal competition network for LINC00511. The miRwalk database was used to find all miRNA targets. Finally, in Fig S3 E, all of the genes in the miRNA-mRNA-LINC00511 axis that could be involved in the ceRNA network were presented.

### B. In-Vitro analysis

#### CRISPR/Cas9-mediated knockout strategies targeting the LINC00511 lncRNA

**Fig. 2:**
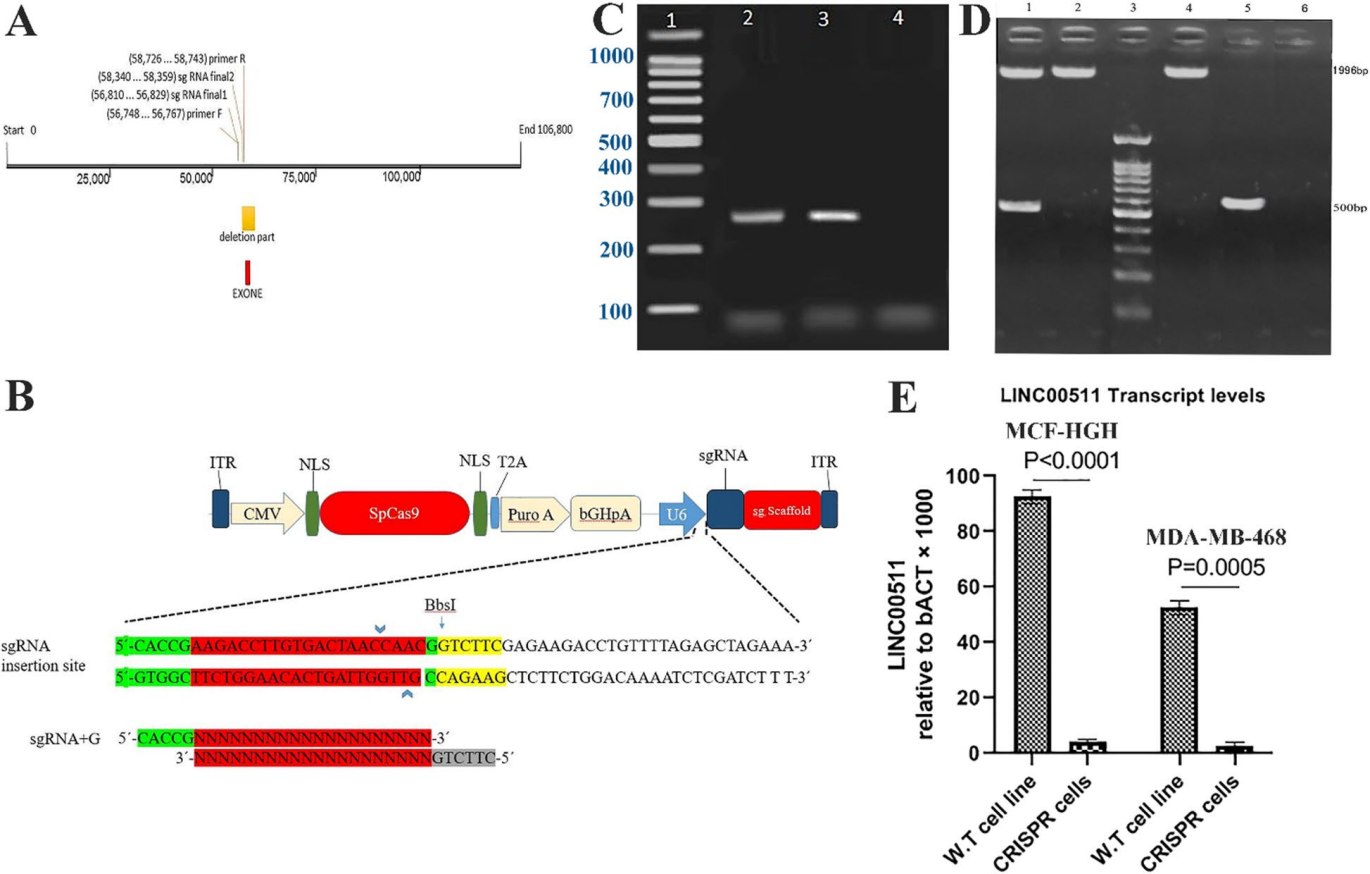
**A **LINC00511 gene schematic, gene length, sgRNA binding site, and gene primers. The position of the fragment deleted by the CRISPR / Cas9 technique is depicted in orange in this diagram. **B** expression portion of sgRNA in vector PX459. The BbsI enzyme is used to form a sticky end, which is then supplemented by sgRNA tags. **C** sgRNAs 1 and 2 results in the Px459 vector. No. 1 is a 100 bp DNA ladder. Nos. 2 and 3 PCR reactions were performed on recombinant vectors PX459-sgRNA1 and PX459-sgRNA2, respectively, yielding 276 bp bands. Negative PCR control (no. 4) (without DNA). **D** Column 1: Heterozygous cells with only one LINC00511 gene allele degraded and two bands 1996 and 480 visible. 2: Control group (PX459-control), in which just one band of 1996 pairs are transfected and the blank vector is transfected. This value indicates that the LINC00511 gene hasn't been degraded. Column 3 100-bp marker. Column 4: No vectors have been transfected blank control cells, and the 1996 band shows the pair. Column 5: Cells that have lost both alleles of the target gene and contain a small band of roughly 480 kb. Column 6: Negative control (no DNA in any of the PCR reagents). **E** LINC00511 transcript levels in MCF-HGH and MDA-MB-468 breast cancer cells compared to GAPDH levels in wild-type and "CRISPR " knockout cells

Our purpose was to disturb LINC00511's proper activity by deleting a region of the LINC00511 gene that was essential for the LINC00511's secondary structure, or by inserting a transcription termination signal into the LINC00511 locus to promote early transcription termination. The first technique, dubbed "CRISPR ablation" here, entailed utilizing two sgRNAs to eliminate a genomic segment precisely (Fig. 2A). We employed two sgRNAs to guide Cas9's endonuclease function to either side of the LINC00511 exon in this method (Fig. 2B). Plasmid amplification in E. coli top10f was accomplished after the production of the pSpcas9-sgRNA 1 and pSpcas9-sgRNA2 vectors. Figure 2C showed PCR results by using vector-specific primers on bacteria bearing the pSpcas9-sgRNA1 and pSpcas9-sgRNA 2 vectors. 276 bp bands in pSpcas9 vectors showed the presence of sgRNA1 and sgRNA2. The presence of sgRNA1 and 2 in vector pSpcas9 was confirmed by the 276 bp bands in lane 2 and 3. Only one allele of the LINC00511 gene was eliminated in heterozygous cells, resulting in two bands 1996 and 480 bp. The 1996 band depicted the pair of blank control cells in which no vector was transfected (Fig. 2D). Also, PCR amplification of wild type (w.t) and knockout alleles in different clones produced by different CRISPER approaches (CRISPR-excision, CRISPR-HDR and CRISPR du-HITI) was shown in Figure S2.

MCF-HGH and MDA-MB-468 cell lines were employed for this study. We acquired 50 MCF-HGH clones during a first round of transfection and screening. 10 clones had one copy of LINC00511 removed, and no clones were homozygous for this ablation, according to PCR from DNA Template. After transfection and screening, we were able to identify 2 out of 50 clones that were homozygous knockdowns for LINC00511, as confirmed by PCR assay of DNA Sample and sequencing of the PCR result (Fig. 2E).

**Fig. 3:**
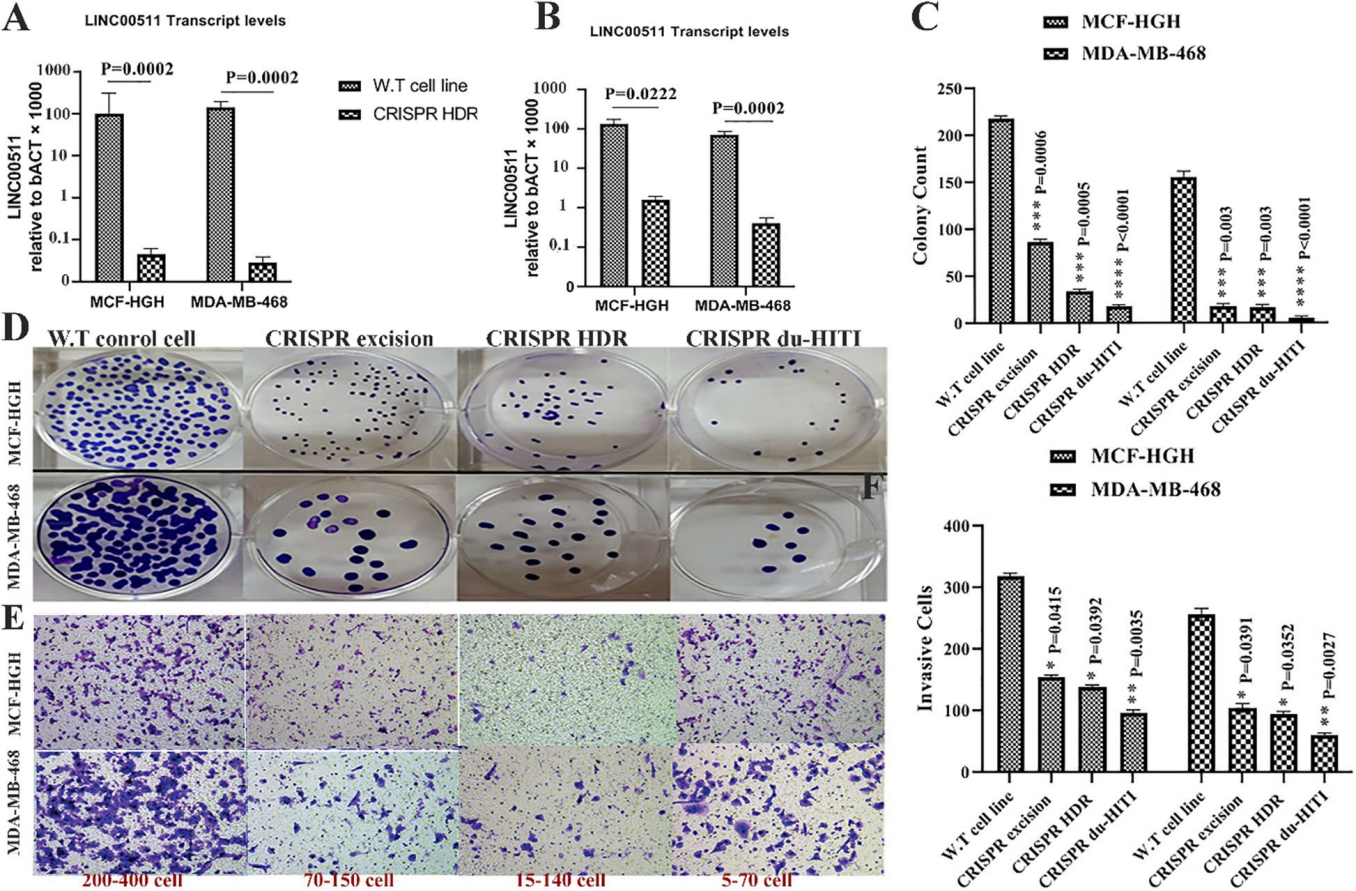
The knockout of LncRNA LINC00511 Suppress malignant cell proliferation and invasion in vitro. **A, B** In wild-type and "CRISPR HDR" knockdown MCF-HGH and MDA-MB-468 cancer cells, LINC00511 transcript levels were compared to GAPDH levels. Based on the relevant standard curves, the absolute copy number for LINC00511 and GAPDH transcripts were measured, and the amount of the LINC00511 transcript was multiplied by the amount of the GAPDH transcript was displayed for three samples of cell line cDNAs. The Mann–Whitney U test is used to examine the statistically significant differences between the wild-type and knockout cell lines. **C, D** MCF-HGH and MDA-MB-468 cells transfected with sh-LINC00511 or sh-NC colony formation test. MCF-HGH and MDA-MB-468 cells transfected with sh-LINC00511 or sh-NC were shown to proliferate in the CCK-8 test. **E** and **F** The number of MCF-HGH and MDA-MB-468 cells that have been invaded. **** *p*-value < 0.0001, *** *p*-value < 0.001, ** *p*-value < 0.01, and * *p*-value < 0.05

Using a simple sgRNA and a donor plasmid with homology arms, the second approach, dubbed "CRISPR HDR," involved inserting a reporter gene and a transcription termination marker in the LINC00511 chromosomal region. We focused on the first few areas of the exon because it was observed that this domain acted as a promoter for exon-derived transcripts. Exon contained a transcription termination marker, which guaranteed that transcripts coming from the proximal portion of the exon were terminated prematurely. To aid in the screening of suitably transformed cells, our reporter construct comprised a green fluorescent protein (GFP) gene as well as DNA for puromycin dihydrochloride tolerance (Fig. 3A).

The "CRISPR du-HITI" technique includes two sgRNAs is used for removing one part of chromosome containing LINC00511 exon and 2 donor plasmids lacking homology arms to introduce two reporters and transcription termination markers.

The donor plasmids employed in these techniques were easier to clone than those used in "CRISPR HDR," and the existence of a selection enabled screening of appropriately changed cells more realistic than in "CRISPR excision." Furthermore, by selecting for both GFP and puromycin, this method could almost always detect homozygous ablation of the target DNA. The GFP sensor will be present in one allele of the chosen clones, while the PuroR sequence will be present in the other. Additionally, huge genomic regions may be deleted and replaced with various reporter cassettes using this technique. We were able to get 6 clones of MCF-HGH and 8 clones of MDA-MB-468 cells with persistent green fluorescence pattern and puromycin resistance during the first trial of transfection, single-cell isolation, and clonal expansion. PCR tests confirmed the knock-in, revealing the introduction of the required segments (Figure S2). When compared with the control cell line, these mutants displayed statistically significant reduced LINC00511 expression levels (29-fold reduction) (Fig. 3B).

### The knockout of LncRNA LINC00511 Suppress malignant cell proliferation and invasion in vitro

Colony formation assay and CCK-8 assay revealed that LINC00511 silencing inhibited the proliferation of MCF-HGH and MDA-MB-468 cells, while it enhanced LINC00511 expression promoted the proliferation of MCF-HGH and MDA-MB-468 cells (Fig. 3C, D). The transwell invasion assay analysis found that LINC00511 silencing reduced the number of invaded MCF-HGH and MDA-MB-468 cells, while it enhanced LINC00511 expression produced the opposite effect (Fig. 3E, F). Consequently, the knockdown of LncRNA LINC00511 inhibited the proliferation and invasion of MCF-HGH and MDA-MB-468 malignant cells in vitro.

### LncRNA LINC00511 is involved in the maintenance of the breast cancer CSC trait

**Fig. 4:**
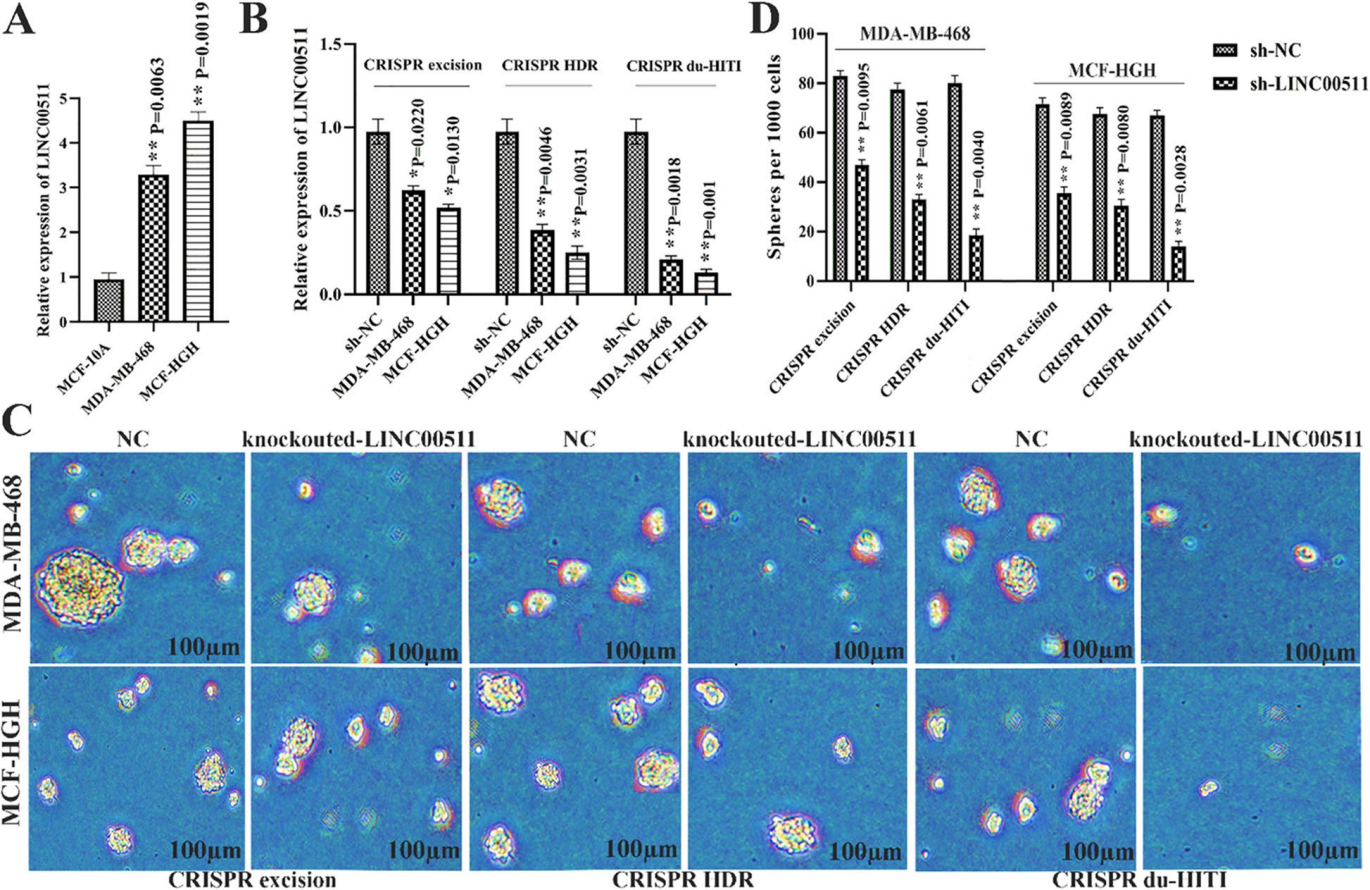
LncRNA LINC00511 has a role in preserving the breast cancer CSC trait. **A** MDA-MB-468 and MCF-HGH breast tumor cell lines, as well as MCF 10A normal cell lines, showed LINC00511 expression. **B** expression of LINC00511 in the MDA-MB-468 and MCF-HGH breast tumor cells. **C** When LINC00511 shRNA was transfected, the mammosphere width and amount decreased. **D** When improved LINC00511 plasmids are transfected, mammosphere amount decreases. ** *P*-value l < 0.01, * *P*-value < 0.05

LINC00511 activity was discovered to be over-expressed in human tumor cell lines (MDA-MB-468, MCF-HGH) compared to MCF-10A control lines using RT-qPCR (Fig. 4A). shRNA targeted LINC00511 and improved expression vectors were introduced into MDA-MB-468 and MCF-HGH human breast tumor cells (Using the 3 CRISPR strategy) to decrease or increase expression (Fig. 4B). Based on the findings of the current study, Silencing of LINC00511 decreased stem factor expression in MDA-MB-468 and MCF-HGH cells. Silencing of LINC00511 decreased mammosphere dimension and frequency in a sphere-formation experiment (Fig. 4C, D). The findings revealed that LINC00511 assisted in the preservation of breast cancer CSC features, implying that LINC00511 played a role in breast cancer cell stemness.

### Nanog expression was boosted by E2F1 at the transcriptional level

**Fig. 5:**
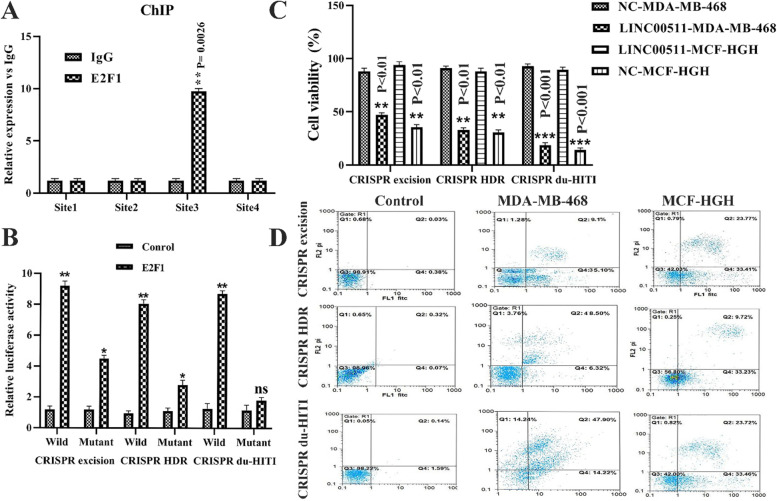
Nanog expression was boosted by E2F1. **A** chromatin immunoprecipitation (ChIP) test was used to determine which area of the Nanog promoter site was the most efficient binding site. **B** The luciferase reporter vector with wild type or mutant Nanog promoter domain sequences (− 586 ~  − 576) was created, as well as the luciferase activity of wild type and mutant Nanog promoter domain sequences. **C** The growth indices (PI) of three different groups were analyzed using a diagram. After 72 h, PI in the CRISPR du-HITI group was significantly lower than the blank control group (** < *P*0.001). **D** Evaluation of the cell apoptosis by Annexin V-FITC staining. Flow cytometer analysis of the apoptotic and necrotic cells. Q1: Necrotic percentage, Q2: late apoptotic percentage, Q3: Live percentage, and Q4: early apoptotic percentage. LINC00511 Knockout enhances apoptosis rate in MDA-MB-468 and MCF-HGH cells

The Nanog gene's promoter was divided into four regions (Table S2). Endogenous E2F1 binds to the (Table S3) Nanog promoter sequence positions (− 1000 ~  − 400) according to a chromatin immunoprecipitation (ChIP) experiment (Fig. 5A). Then, by using bioinformatics techniques, the linkage points of E2F1 for promoter Nanog is predicted.

By utilizing PROMO and JASPAR, we discovered that transcription factor E2F1 could interact with the promoter site of the Nanog gene (+ 100 ~  − 2000 nt). According to our findings, the non—transgenic alleles of Nanog promoter sequences had more luciferase activity than the recombinant typ. This indicated that the binding site was located within the E2F1 and Nanog promoter sequences (Fig. 5B). The researchers discovered that E2F1 increased Nanog expression at the transcriptional level, suggesting that the LINC00511/E2F1 axis boosted breast cancer stemness and carcinogenesis.

### CRISPR/Cas9-mediated Knockout of LINC00511 increased apoptosis rate using suppress apoptosis-related genes

MTT was used to measure the effect of LINC00511 knockdown on MDA-MB-468 and MCF-HGH cell growth per 72 h. The growth rate of MDA-MB-468, MCF-HGH breast cancer cells was lowered in all CRISPR techniques (P < 0.001), and there was a significant difference between the control and mutant groups' growth rates (Fig. 5C). To examine the potential variations in apoptosis across the experimental groups, an Annexin V-PI staining technique was used. Figure 5D depicted the percentages of early, late, necrosis, and surviving cells. The rates of early apoptosis, late apoptosis, and necrosis were less than 5% in the blank control cells of three CRISPR procedures. In control groups, the number of live cells rats was more than 95%. Apoptosis rates in MDA-MB-468 cells were 57.18, 42.95, and 57.18% for CRISPR excision, CRISPR HDR, and CRISPR du-HITI, respectively. CRISPR excision, CRISPR HDR, and CRISPR du-HITI induced 44.2, 54.82, and 62.12% apoptosis in MCF-HGH cells, respectively (Fig. 5D).

Cell cycle advancement was pertinent to the acceleration of cell proliferation. Flow cytometry was used to examine cell cycle regulation in the CRISPR excision, CRISPR HDR, and CRISPR du-HITI group of cells. LINC00511 knocked out cells with an increase in G0.G1 and a decrease in the S phase to G2.M phase ratio compared to the control groups. LINC00511 knockout hindered cell cycle progression, according to these findings.

**Fig. 6:**
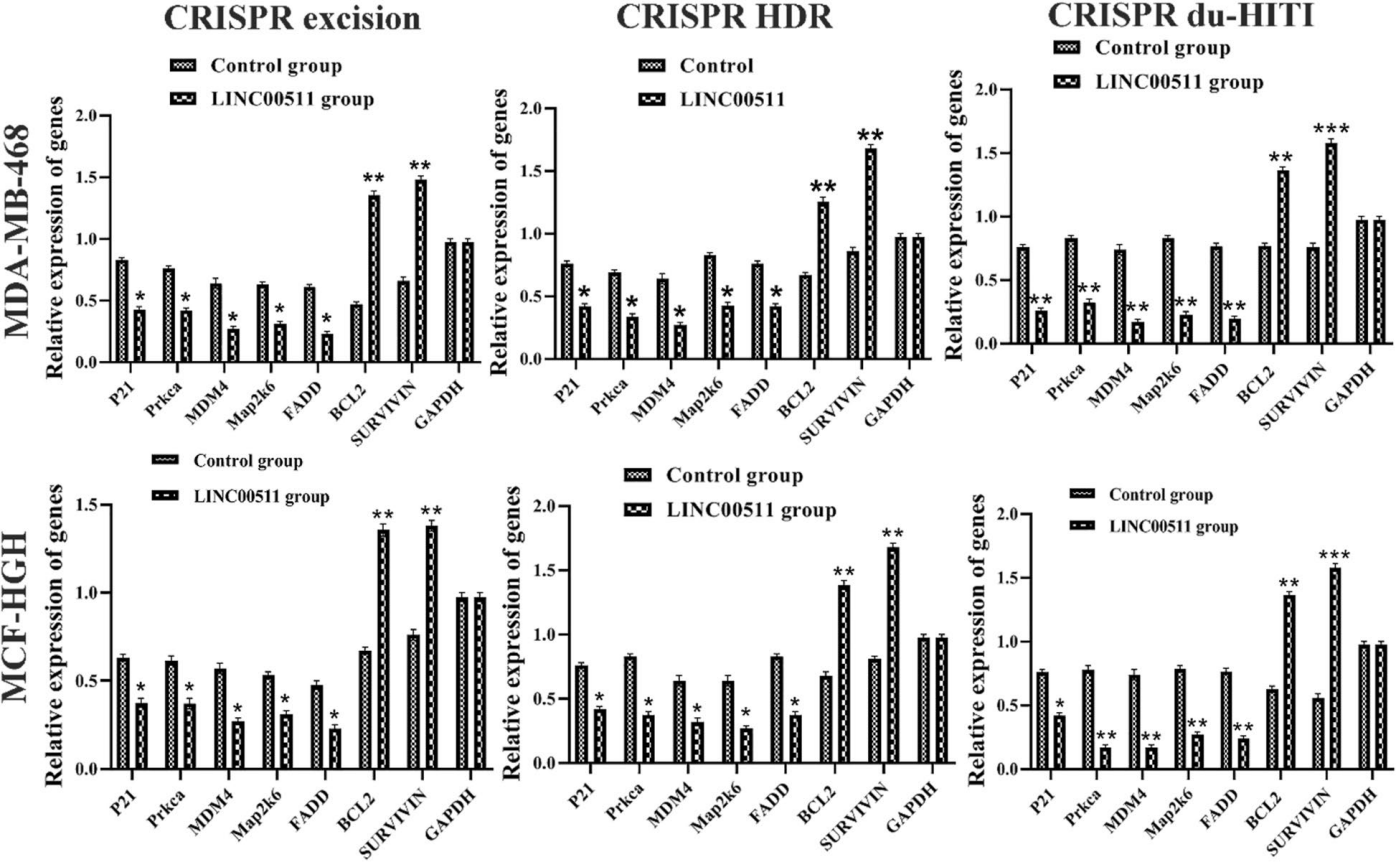
Knockout of LINC00511 gene in MDA-MB-468 and MCF-HGH cell line leads to induction of expression of pro-apoptotic genes P57, P21, Prkca, MDM4, Map2k6, and FADD at a significant level ** *P* < 0.01 and reduction of expression of anti-apoptotic genes *SURVIVIN* and *BCL2* at a significant level *** *P* < 0.001. Data were normalized by the *GAPDH* reference gene. There was no significant difference in the expression of pro/anti-apoptotic genes in the Blank control groups

Proapoptotic genes P57, P21, Prkca, MDM4, Map2k6, and FADD, as well as antiapoptotic genes BCL2 and SURVIVIN, were chosen for the current study, and their expression was measured by using real-time PCR in two cell lines. The deletion groups had increased proapoptotic gene expression than the blank control group significantly (Fig. 6A, *p* < 0.01). Afterwards, we examined the expression of anti-apoptotic genes (BCL2, SURVIVIN) in two separate cell types. Gene expression of BCL2 and SURVIVIN was greater in the control cell line than in knockout cells (*p* < 0.05).

## Discussion

LINC00511 was downregulated in a variety of tumors, and this was linked to survival and metastasis in human cancers [23]. However, the clinical importance of LINC00511 overexpression in human cancers was still under debate, therefore, we used The Cancer Genome Atlas (TCGA) data to conduct a meta-analysis and bioinformatics research to analyze the clinical significance of LINC00511 expression level comprehensively. By the way, we conducted a thorough examination of the biological roles of LINC00511 in malignant tumors, as well as the processes involved. We discovered that greater LINC00511 expression was linked to poorer OS and DFS, as well as a higher incidence of lymph node metastases in this study. The processes behind LINC00511's oncogenic functions were complicated, which may assist to clarify how LINC00511 expression levels in cancers were clinically significant [24]. Most researchers found that LINC00511 might boost cancerous cell aggressiveness by sponging miRNAs and modifying the production of their targets, such as miR-185-3p/E2 F1/Nanog [18], miR-625/CCND1 [25], miR-524-5p/YB1/ZEB1 [26], miR-124-3p/EZH2 [27], and miR-765/APE1 [28].

The results of this investigation revealed that lncRNA LINC00511 production was higher in breast cancer cell lines, which might be linked to a worse outcome. These findings implied that LINC00511 was a breast cancer regulator. The effects of LINC00511 on the functional morphology and stemness of breast cancer cells were examined. Gain and loss-of-functional experiments demonstrated that LINC00511 increased sphere development, stem factor production, and contributed to the preservation of breast cancer CSC characteristics, implying that LINC00511 played a role in tumor cell differentiation [29]. CSCs were previously identified from breast cancer, lung malignancy, colon cancer, malignant melanoma, and glioblastoma [26]. Cancer invasion and metastasis were critical carcinogenic biological characteristics. CSCs were implicated in tumor invasion and metastasis in several studies [30, 31]. The strategy by which LINC00511 controlled breast cancer pathogenesis was studied further in experiments. E2F1 interacted with the Nanog gene promoter, indicating that it acted as transcriptional activation. The direct action of E2F1 and Nanog was validated by using a luciferase reporter experiment, ChIP, confirming that the regulatory pathway was LINC00511/E2F1/ Nanog. In bladder cancer and colorectal cancer, the transcription factor E2F1 was discovered as an oncogenic component [32]. E2F1 was also discovered to be involved in the production of Nanog in breast cancer and to be up-regulated and engaged in the tumorigenesis of breast cancer.

As more lncRNAs were discovered, functional investigations were becoming more important in determining their biological functions. Approaches aimed at inhibiting lncRNA expression were important for understanding the biological roles of lncRNA, just as they were for protein-coding genes. However, for lncRNAs with nucleus localization, the two commonly used techniques, RNA interference, and antisense approaches, were ineffective [33]. Furthermore, inadequate target gene depletion, off-target effects, and technical variances were all related to these approaches. An excellent option was to use recently emerging genome editing technologies to change the genomic location of an lncRNA such that the linked lncRNA became non-functional [34]. Moreover, because lncRNAs were non-coding, alterations at the associated genomic regions must be substantial enough to have an unfavorable impact on the development and biological function of the expressed RNA. A simple excision followed by NHEJ, for example, could not generate enough structural alterations in a specific lncRNA [35]. Three alternative CRISPR-mediated KO techniques were employed such as: "CRISPR excision," "CRISPR HDR," and "CRISPR du-HITI" to modify the LINC00511 location in distinct breast cancer cell lines.

The use of two sgRNAs to remove a genomic region of lncRNA genes were described before [36, 37]. There were no donor plasmids employed in the implementation of this model, and hence no reporter sequences were put into the genome. As a result, selecting Knockout cells became a time-consuming process. "CRISPR HDR," on the other hand, employed donor plasmids, allowing the user to choose the molecular targets based on fluorescence and/or resistance to antibiotics [38, 39].

However, homology elements must be cloned into the donor plasmid for this technique to work. The "CRISPR du-HITI" method, which does not depend on the existence of homologous bands, made it possible to select cancer cells. This method allowed us to control Knockout production so that each cell exhibited both resistance to antibiotics and fluorescence characteristics may be easily identified as a dual allele Knockout. Genomic PCR, sequencing, and RT-qPCR were used to confirm the LINC00511 Knockout cells produced in this investigation. We didn't expect any activation outputs from "CRISPR excision" and "CRISPR du-HITI" Knockout cells because the qPCR forward and reverse primers were equivalent to an exon. Exon was deleted in each of these procedures. However, in "CRISPR HDR," the first portion of the exon was designated with a reporter/transcription termination signal-containing element. As a result, an amplifying result was anticipated. RNA-seq, soft agar colony formation assay, and MTT analyses were performed on the LINC00511 Knockout cells. Several genes were expressed differently in these cells. Soft agar colony growth and MTT analyses were used to confirm that the LINC00511 Knockout cells had a limited ability to proliferate. This conclusion was consistent with prior observations that LINC00511 inhibition influenced genes associated with cell growth regulation.

## Conclusions

To summarize, we described three CRISPR/Cas9-mediated techniques that were useful for lncRNA functionality assessments. Additionally, the LINC00511 Knockout cell lines developed in this work could be utilized for further analytical investigations to disclose the full range of LINC00511 activities. The du-HITI approach elaborated in this paper was simple to use and might be used to generate homozygous individuals' knockout for a particular lncRNA of concern.

## Methods

### A. Bioinformatics analyzes

#### Search strategy and study selection

We perused suitable and related papers in PubMed, EMBASE, Web of Science and Wanfang from February to June 2021. 'Long intergenic noncoding RNA 00,511' or 'LINC00511' were used as search phrases. The papers that meet certain requirements were all included at the same time: 1) Participants were divided into two groups based on their level of LINC00511 transcription; 2) Risk ratio with 95 percent confidence levels (CIs) for the connection relationship between LINC00511 transcription and survival rates was accessible; and 3) articles were published in English. The following were the contraindications: 1) research studies that were letters, conference abstracts, meta-analysis, or review papers; 2) research studies that examined the prognostic significance of LINC00511 transcriptional level using The Cancer Genome Atlas (TCGA) datasets; or 3) research studies that recruited the same participants.

### Extraction of data and quality evaluation

Data from qualifying investigations were retrieved by two writers separately. Any discrepancies were resolved by discussion among all of the writers. First author, date of publication, location, cancer type, therapy category, statistical significance, detection technique, reference standards, and cut-off parameters, HRs for OS with 95 percent confidence intervals, logistic regression type, and follow-up length were all retrieved. When HRs were obtained by using both univariate and multivariate evaluation, the multivariate HRs were chosen first since they had fewer confounding variables. HRs with 95 percent CIs would be generated from Kaplan–Meier surviving graphs using the Engauge Digitizer program if not explicitly supplied in the article. The Newcastle–Ottawa scale (NOS) parameters, which were a star grading system from 0 to 9, were used to assess the qualitative characteristics of investigations [40].

### Validation through the use of publicly available data

The Cancer Genome Atlas (TCGA) publishing criteria were applied in this work. The connections of LINC00511 transcriptional level with OS and DFS were assessed by using Gene Expression Profiling Interactive Analysis (GEPIA). The K-M technique and log-rank assessment were used to compute the logistic regression, and the HRs and *p* values were displayed in the K-M curve diagrams [41].

### Identification of differentially expressed lncRNAs and co-expression of lncRNA and apoptotic genes

In order to investigate the role of aberrant expression of lncRNAs in breast cancer, a bioinformatics approach was used to predict the differentiallyexpressed genes. The symbols and names of the lncRNA genes were obtained from the database of the HUGO Gene Nomenclature Committee (genenames.org). The breast invasive carcinoma dataset was obtained from The Cancer Genome Atlas (TCGA) at the cBioPortal for Cancer Genomics, consisting of 15,278 samples (cbioportal.org). By selecting the cancer study and genomic profiles, the genes of lncRNAs that have been downloaded may be entered and the information submitted. To investigate the co-expression of lncRNA and apoptotic genes that is highly expressed in breast cancer, initially all the genes involved in the apoptosis pathway were obtained from the site QIAGEN (www.qiagen.com) and it was done in the way that lncRNA expression was evaluated in breast cancer. The lnc expression network and the apoptotic genes that showed high expression were plotted.

### Identification of differentially expressed lncRNAs and miRNAs

In order to investigate the role of aberrant expression of lncRNAs in breast cancer, a bioinformatics approach was used to predict the differentiallyexpressed genes. The symbols and names of the lncRNA genes were obtained from the database of the HUGO Gene Nomenclature Committee (genenames.org). The breast invasive carcinoma dataset was obtained from The Cancer Genome Atlas (TCGA) at the cBioPortal for Cancer Genomics, consisting of 1,105 samples (cbioportal.org). By selecting the cancer study and genomic profiles, the genes of lncRNAs that have been downloaded may be entered and the information submitted. miRNAs may be selected in the same way.

### lncRNA-miRNA interaction analysis

In order to identify the miRNAs which were able to target lncRNAs, the binding of lncRNAs to miRNAs (including the folded RNA predicted structure of the lncRNAs and miRNAs) was predicted by using the bioinformatics tool RegRNA 2.0 (regrna2.mbc.nctu.edu. tw/detection.html). The protein sequence was obtained from the GenBank database of the National Center for Biotechnology Information (NCBI; ncbi.nlm.nih.gov/genbank). In addition, when predicting the miRNA target sites, the minimum folding free energy was set under < -20 and the system score was set to > 160. An increased score indicated a stronger binding ability. Due to the large number of lncRNAs which were differentially expressed in breast cancer, the lncRNAs selected fell above 3% alteration frequency. Additionally, the lncRNA sequences associated with *Homo sapiens* were searched in advance by using the NCBI database.

### miRNA target prediction and construction of the lncRNA-miRNA-mRNA network

In order to predict which genes to target with screening miRNAs of mirtarbase databases (http://mirtarbase.mbc.nctu.edu.tw/php/index.php) it was used Genes which were identified by using of this database.

In addition, it was confirmed that the lncRNAs exhibited High expression (LINC00511) to maximize the clarity of the network diagram. The lncRNA-miRNA-mRNA interaction network was constructed by using Cytoscape software (version 3.7.1; http://www.cytoscape.org/download.php). Apoptotic genes were searched in this network apart from the expression network.

## Gene ontology (GO) analysis

In order to further investigate the biological effects of aberrantlyexpressed lncRNAs and miRNAs in breast cancer, GO enrichment of the target gene were carried out using the GOrilla tool (cblgorilla.cs.technion. ac.il). For each GO term, a list of associated genes is returned with the most optimal at the top of the list. Each gene name is specified by the gene symbol and followed by a short description of the gene.

### B. In-vitro analyzes

#### Cell culture

The cells were obtained from Iran's National Cell Collection (Pasteur Institute, Iran) and grown according to ATCC guidelines. Two different breast adenocarcinoma cells were employed. At 37 °C in 5% CO2, MCF-HGH and MDA-MB-468 cells were cultivated in Dulbecco’s modified Eagle’s medium (DMEM; Gibco) enriched with 10% FBS (Gibco, USA), 50 U/ml penicillin, and 50 μg/ml streptomycin (Sigma-Aldrich, USA). Normal human breast epithelial cell (MCF-10A) was employed as the Control group.

### DNA constructs and gene targeting

CRISPR/Cas9 technology was utilized to knockout the *LINC00511* gene in the human breast cancer cell lines. The LINC00511 gene sequence was found in the GenBank sequence collection of the National Center for Biotechnology Information (National Biosciences, Inc., Plymouth, MN). The CRISPR specially developed CHOPCHOP website (https://chopchop.cbu.uib.no) and (http://crispr.mit.edu/) were used to generate single guide RNA (sgRNA) patterns targeting distinct portions of the LINC00511 gene.

Three vectors were generated: pX459 (involving U6 promoter-sgRNA insertion site-sgRNA scaffold and CAG promoter-Cas9-T2Apuromycin N-acetyltransferase gene-bovine growth hormone polyadenylation signal), pX460–1 (involving U6 promoter-sgRNA insertion site-sgRNA scaffold and CAG promoter-enhanced GFP (EGFP)-bovine growth hormone polyadenylation signal), and pX461–1 (involving U6 promoter-sgRNA insertion site-sgRNA scaffold, and CAG promoter-puromycin N-acetyltransferase (PuroR)-bovine growth hormone polyadenylation signal) were used for sub-cloning of sgRNAs. To achieve this, nucleotide sequences with the sgRNA generating region and steaky ends (Table 1) were produced. To achieve this goal, nucleotide sequences (Table 1) with the sgRNA encoding pattern and steaky ends were produced (Macrogen Inc., South Korea), annealed, phosphorylated, and cloned into BbsI-digested and gel isolated vehicles (using Gel Extraction Kit; DENAzist Asia Co., Iran). The PAM region was also included after the sgRNA coding segment in "CRISPR du-HITI" approaching plasmids. The left homologous arm (546 bp), DsRed2, herpes simplex virus thymidine kinase polyadenylation signal, CMV promoter, PuroR, IRES2, EGFP, SV40 polyadenylation signal, and right homologous arm (546 bp) were all included in the plasmid used for "CRISPR HDR" addressing (832 bp). Table S1 shows that constructs (verified by Sanger sequencing; Macrogen Inc., South Korea) were being used for transfection of breast tumor cell lines using Lipofectamine 2000 chromophore (Thermo Fisher Scientific, USA). Each culture (50 colonies) was observed to proliferate before being chosen by PCR assay two weeks after transfected for "CRISPR excision" and elimination of the LINC00511 exon. The GFP expressing and puromycin dihydrochloride tolerance of cultures was used to qualify them for "CRISPR du-HITI" and "CRISPR HDR" (Sigma-Aldrich, USA). Each cultures colony of "CRISPR excision," "CRISPR du-HITI," and "CRISPR HDR" were evaluated using genetic Material, PCR detection, and Genome Sanger sequence assay (Macrogen Inc., South Korea).

**Table 1:** List of specific primers used in this research

*Gene*	*Primer*	*Sequence* *5’–––––––––––3’*	*TM(°C)*
*hU6*	Hu6-F	GAGGGCCTATTTCCCATGATT	62
*PX459-sgRNA1*	P-LINC001-sg1	AAGACCTTGTGACTAACCAAC	62
*PX459-sgRNA2*	P-LINC001-sg2	AAGACCCGTCGTTTTTCGAAG	62
*LINC00511*	LINC-F LINC-R	CTCCTGTCACAGCCTCAGTG CTCGGCTGAGTCGCGTTC	62
*P57*	P57-F P57-R	CTTCTTTGACCCTGACACC CTGAACATGGAGAGATAGTGC	59
*Survivin*	Sur-F Sur-R	GAGAACGAGCCAGACTTGG GCTTTCCTTTCTGTCAAGAAGC	62
*BCL2*	BCL2-F BCL2-R	TGTGGCCTTCTTTGAGTTCG TACAGTTCCACAAAGGCATCC	58
*P21*	P21-F P21-R	ACTGTCTTGTACCCTTGTGC CTTCCTCTTGGAGAAGATCAGC	58
*Prkca*	Prkca-F Prkca-R	′CGACTGTCTGTAGAAATCTGG CACCATGGTGCACTCCACGTC	59
*MDM4*	MDM-F MDM4-R	AATGATGACCTGGAGGACTCTA ACTGCCACTCATCCTCAGAGGTA	59
*Map2k6*	Map2k6-F Map2k6-R	AGCGAAACCCTGGCCTTAAA CACACATCACCCTCCCGAAA	58
*FADD*	FADD-F FADD-R	C GGAAATGGGACAAAACATCCT TGCGGGAGTAGTTGGAAAGT	59
*GAPDH*	GDH-F GDH-R	GCCAAAAGGGTCATCATCTCTGC GCCAAAAGGGTCATCATCTCTGC	62
*E2F1*	E2F1-F E2F1-R	TGATTGTGGCAAAGGAGGA CTCTTCCTTGCTCGTTGTTGGTAT	62

### Isolation and analysis of genomic DNA

The DNA extraction Kit (*Cinnacolon*, Tehran, Iran) was used to extract DNA Molecules from both wild-type and knockout cell populations, which was then exposed to PCR analysis. After smooth and reaction recuperation, PCR-amplified products were submitted to Sanger Sequencing technology (Macrogen Inc., South Korea).

### Detection of mismatched duplexes by T7 endonuclease assay

A mismatch-sensitive T7 endonuclease 1 test (New England Biolabs) was used to confirm that DNA cleavage and targeted sequence disruption occurred at the specified spot. DNA was extracted from the cells using the FavorPrep™ GEL Purification and DNA extraction kit (FAVORGEN Biotech Corp-Taiwan, according to the manufacturer's instructions). In different microtubes, 10 μl (200 ng) of each DNA sample was combined with 2 μl of 10X NE-Buffer 2 buffer and 19 μl of nuclease-free water. At 95 °C for 10 min, the samples were heated. Then, it was allowed to cool at room temperature gradually. 19 μl of each sample were combined with 1 μl of T7 endonuclease I (5 units/μl) and incubated at 37 °C for 15 min before being examined on an agarose gel. Band intensities were measured using Tanon-electrophoretic software (Tanon Science & Technology Co., Ltd., Shanghai, China), and the targeted disruption was seen.

### Reverse transcription quantitative PCR

The Y-Tizol RNA extraction Kit was used to extract total RNA from both wild-type and knockout cell populations (Yekta-Tajhiz, Iran). Utilizing capillary electrophoresis and a 2000 Nanodrop spectrograph, the quality, and variety of extracted RNA were determined (Thermo Scientific, USA). With the assistance of random hexamer primers and MMLV reverse transcriptase, 1 μg of total RNA was transcribed reversely and cDNA was synthesized (Thermo Fisher Scientific, USA). Quantitative RT-PCR reactions incorporating Premix Ex-Taq (Probe qPCR) master mix (Takara, Japan), 2 μl cDNA, 500 nM primers, and 100 nM probe (dual-labeled hybridization probes, 5'FAM-3'BHQ1-labeled for LINC00511 and 5'CY5–3'BHQ2 for GAPDH) in a 20 μl reaction mixture were conducted (Qiagen, USA). The following amplifying stages were used: 95 °C for 5 min, then 40 cycles of 94 °C for 30 s, 62 °C for 30 s, and 72 °C for 30 s. Sanger sequencing was used to establish the identification of the PCR products (Macrogen Inc., South Korea). Standard curves were created by subcloning amplified segments and serial diluting. Three PCR assays were run on each concentration, and real-time measurements were taken in duplicate. The log of copy numbers was then compared to cycle threshold (Ct) quantities. Efficiency (E) was estimated for each qPCR reaction based on the computed gradient of standard curves constructed utilizing fivefold serial dilutions vector mixtures, using the following formulae: E = (10–1/slope-1) 100%. In the investigated range, all calibration curves were normal and had a good coefficient of correlation (R2). Based on the corresponding calibration graph, the relative copy number of LINC00511 and GAPDH transcripts was calculated. The amount of the goal transcript (LINC00511) was split by the amount of the reference gene (GAPDH) for 2 different sets of cDNAs, and the results were shown.

### Assay for sphere formation

MCF-HGH and MDA-MB-468 breast cancer cells treated with vectors were plated in six-well plates and incubated (Corning, NY, USA). As previously explained [18], cells (2 × 10^5^) were cultivated in serum-free DMEM media with EGF, hFGF (Peprotech, USA), insulin, and penicillin/streptomycin (Gibco) supplemented. Under a light stereomicroscope, clones of spheroids were fixated and dyed with dye solution (crystal violet), then identified (Olympus, Tokyo, Japan).

### Assay for CCK-8 proliferation and colony formation

The CCK-8 measurement was carried out with the use of a CCK-8 detection kit (Dojindo Japan). The transfected cells were plated into culture plates, and the cell lines were cultured for 10 h before being treated with the CCK-8 chemical. Absorbance was measured at 450 nm.

### Invasion screenings in transwells

Using a 24-well transwell chamber (Corning), breast tumor cells were planted on a member that had been pre-coated with adherent cells (BD Biosciences, San Jose, CA, USA). The individuals on the top surfaces were brushed after 24 h of treatment, and the invaded individuals were fixated with 4 percent paraformaldehyde and labeled with Giemsa. After that, the cells were observed by using optical microscopy.

### Assay for luciferase gene reporter

The wild-type and mutant alleles for the E2F1 interaction of the Nanog promoter region were used to generate luciferase reporter constructs. The Lipofectamine 2000 solution was used to co-transfect the plasmids with E2F1 into MCF-HGH and MDA-MB-468 cells (Thermo Fisher, USA). Dual-Luciferase Reporter Assay Kit (Promega) was used to assess the functionality of the Renilla vector (Promega).

### MTT assay

The cell viability was validated using the MTT cell viability Kit I (Roche, Switzerland) colorimetric test. In a 96-well flat-bottomed plate, 5 × 10^3^ cells/well were plated and cultured at 37 °C in a 5% CO2 incubator. Cell viability was measured over three days (24 h, 48 h, and 72 h), with the cells in each well was being washed twice with PBS. Each well was filled with 100 µl of serum-free media and 5 µg/ml Sigma MTT, which were incubated for 4 h at 37 °C in a CO2 incubator. The medium was gradually eliminated, and DMSO was introduced. The ratio of optical density at 570 nm to the background at 690 nm was measured with a State Fax-2100 ELISA plate reader to detect MTT metabolism to generate blue formazan (Awareness Technology, Palm City, FL).

### Cell cycle analysis

Absolute ethanol was used to fix the cells for 24 h. The cells were washed twice in PBS before being stained for 15 min with BD Bioscience Pharmingen's PI/RNase staining buffer. FACS flow cytometry was used to determine the DNA content of the cell population. FlowJo V10 software was used to evaluate the cell cycle data (Tree Star, Ashland, OR).

### Expression of apoptosis-related genes by quantitative real-time PCR

The expression of the proapoptotic genes P57, P21, Prkca, MDM4, Map2k6 and FADD, as well as the antiapoptotic genes *BCL2* and *SURVIVIN*, was assessed by using a quantitative real-time PCR technique with SYBR green detection. Quantitative real-time PCR was carried out utilizing specific primers (Table 1) and a SYBR® Premix Ex Taq™ II kit (TaKaRa, Japan) based on the manufacturer’s instructions. Relative gene expression levels were quantified by normalizing the respective GAPDH level. Experiments were conducted in duplicates.

### Statistical analysis

All studies were done in duplicate and the results were reported as mean SD. One-way ANOVA and independent samples t-tests were used to analyze the differences between groups. SPSS software was used to conduct the analysis, and Graph-Pad Prism was used to create the graphs. Significance was defined as a *P* value less than 0.05.

## Supplementary Information

**Additional file 1.**

## Data Availability

Data and materials are available by authors.
