# Supplementary material for: CRISPR/Cas9-mediated LINC00511 knockout strategies, increased apoptosis of breast cancer cells via suppressing antiapoptotic genes
Source: Biol Proced Online. 2022 Jul 5;24:8. doi: 10.1186/s12575-022-00171-1 (PMC9254607; doi:10.1186/s12575-022-00171-1)
Supplement: Supplementary file 1 — Additional file 1. [file 12575_2022_171_MOESM1_ESM.docx]

**SUPPLEMENTARY FILES**

**Fig. S1** Flow diagram of the bioinformatic study selection.

The results of bioinformatics analysis also showed that mutation frequency and copy number change (CNV) were different for apoptosis-related genes in breast cancer (Fig S3 A). The pathways of apoptotic genes that correlated with LINC00511 were plotted using clueGo in Fig S3 B. All apoptosis genes were identified based on the QIAGEN database that correlated with LINC00511 (Fig S3 C).The L network was then displayed for LINC00511. Based on the L database, all genes associated with the mice identified for LINC00511 were extracted and displayed in Fig S3 D. The prkca gene was the only gene present in both the L network and in express expression correlation with LINC00511.RNA internal competition network for LINC00511 is displayed.


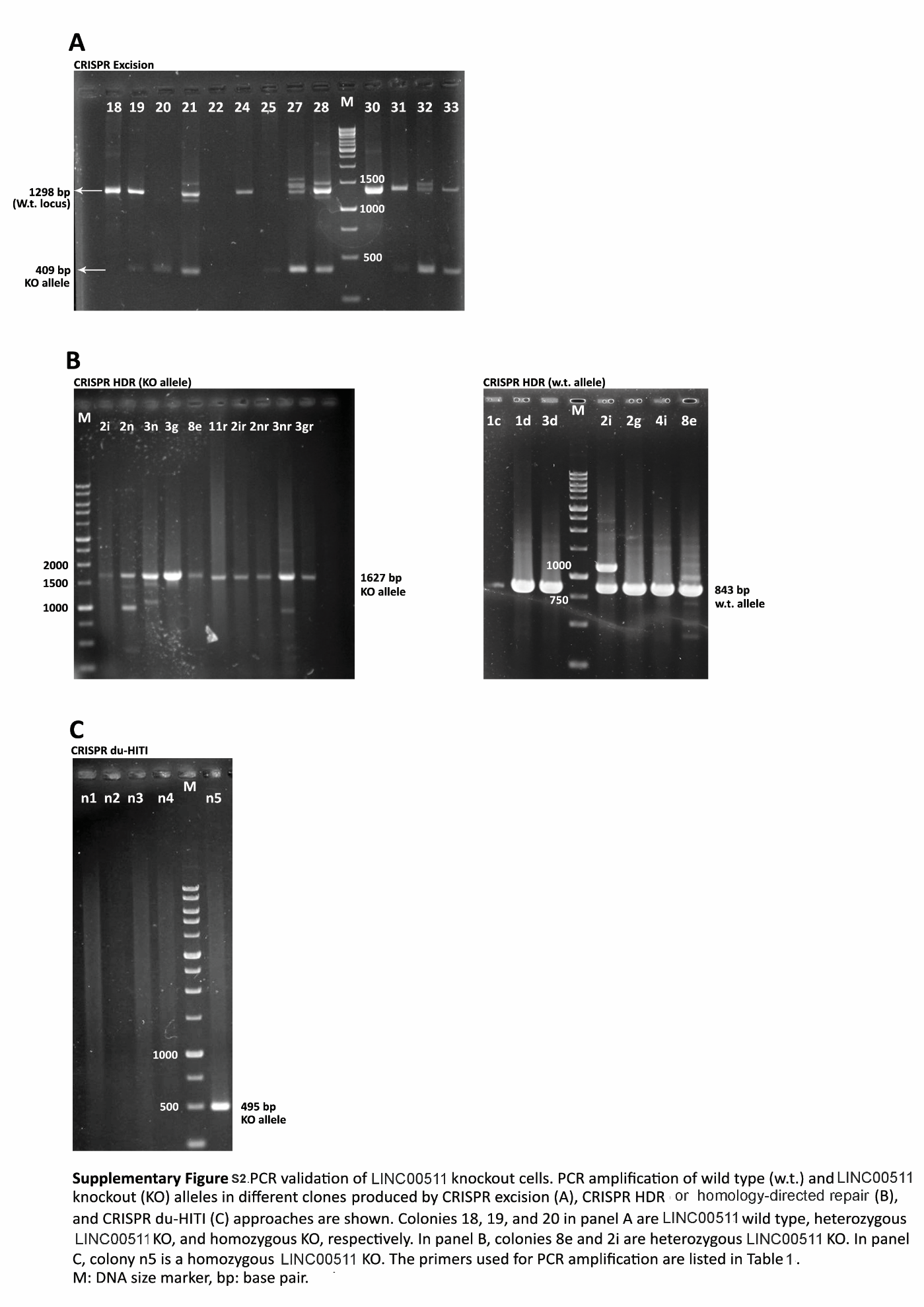


| 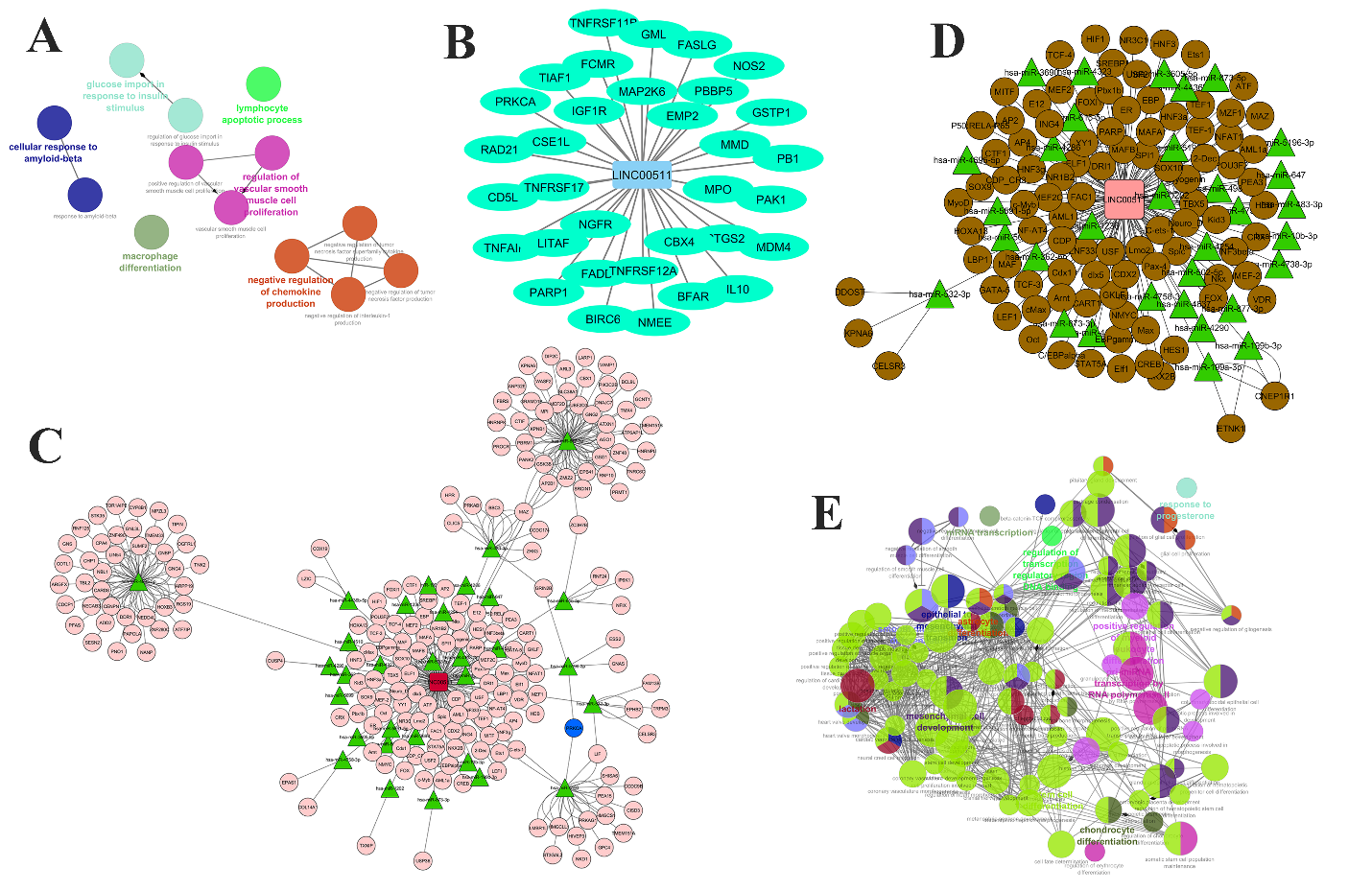  **Fig S3.** **A** The pathway of apoptotic genes that correlated with LINC00511 is displayed using clueGo. The genes are enriched based on the KEGG database. **B** All apoptosis-related genes are shown in the QIAGEN database, which correlates with LINC00511. **C** The L network is displayed for LINC00511. Based on the L database, all genes associated with the mice identified for LINC00511 have been extracted and displayed. The prkca gene was the only gene present both in the L network and in expression correlation with LINC00511 (marked in blue). **D** RNA internal competition network for LINC00511 is displayed. All miRNA targets are identified based on the miRwalk database and shown. **E** All pathways of genes located in the miRNA-mRNA-LINC00511 axis can play a role in the ceRNA network. All genes in the ceRNA network were enriched and displayed in the Cytoscape based on the ClueGo program. |
| --- |

**Table S1** The DNA constructs that were employed in this work.

| **Construct** | **Features** | **Application** |
| --- | --- | --- |
| pX459-1 | hU6 promoter- sgRNA (downstream of LINC00511 Exon  1)-sgRNA scaffold-CAG promoter-Cas9-T2A- PuroR-bGH polyA | CRISPR Excision  CRISPR du-HITI |
| pX459_2 | hU6 promoter- sgRNA (upstream of CCAT1 Exon  1)-sgRNA scaffold-CAG promoter-Cas9-T2A- PuroR-bGH polyA | CRISPR Excision  CRISPR du-HITI |
| pX460_11 | hU6 promoter-sgRNA (downstream of LINC00511 Exon) plus PAM-sgRNA scaffold-CAG  promoter-EGFP-bGH polyA | CRISPR du-HITI |
| pX461_11 | hU6 promoter-sgRNA (downstream of LINC00511 Exon) plus PAM-sgRNA scaffold-CAG  promoter-PuroR-bGH polyA | CRISPR du-HITI |

**Table S2**. Primers sequences for ChIP.

|  | Sequences 5’-3’ |
| --- | --- |
| Site 1 | forward, 5’-ACCTTCCGCCTGACACCTTTGC-3’  reverse, 5’-TCGGCGGCCTCAACAATGG-3’ |
| Site 2 | forward, 5’-TAGATCAGAATAGTCAATGGTGGA-3’  reverse, 5’-AACAAATGTTTTAGCATTGGGATCT-3’ |
| Site 3 | forward, 5’-TGTGGCAGAAAGGATTGGA-3’  reverse, 5’-TTGCAGGGTCATCATCAACG-3’ |
| Site 4 | forward, 5’-CTCGTACCAGGCGAAAAAAG-3’  reverse, 5’-ACCAGAGGGGACACAGTACA-3’ |

**Table S3** The promoter region of Nanog gene (-2000~0).

| Transcription factor E2F1 binds with the sites of Nanog gene promoter at -586~-576 (TCTCCCGC). >hg19_knownGene_uc009zfy.1 range=chr12:7939995-7948755 5'pad=0 3'pad=0 strand=+ repeatMasking=none |
| --- |
| CGCAAAGTGCTGGGATTACAGGTGTGAGCCACCGTGCCCAGCCGTTAGCT -2000  CATTTTAACACATCCTTAGTCCAGCCTGTTCCAAAAAATCTAAAGTCAGA  TAGCTTCCTAAACCTCAACTTTATTCCAATTGCTTTCCTTGGCGAAGAAT -1900  GTAGTAAGTCGGCCTTCCAGCCACCAGCCCCTTCCCTTTGGTCTTTCACT  CCGGAGGCTCTTACCCTAGACACAATGGGACAGGGAGCGGGGGATGGGGG -1800  AATTCAGCTCAGGCTTTTATGCAAAGACCCCCTTCTGCAAAGAACAAAGC  TTCTGGTACCTGCCCTTTGGAGAGCTGCGGGCAAGCTCAGCCTCGGTGAG -1700  TCTTGGTGGCCTTGACAGCCCCCACTTAACAAACTGTGCTGATTAAGAGA  GACAGGAGGGCAAGTTTTTCCCTTCTTTTAAAGAAATCATCCTATTTCCT -1600  ACGAGACATAGACTATCTGCCTGAAGCATGATGTACTAGCCCCACTCACC  GGCTCCCTGATGCCCCTATGCTTAATCTTCTCCGGAATGGTAGTCTGAGA -1500  AGAAAAAAGATTACGCCCAATTTCATTTCCTTGTTTCACATCAAGCAATA  CTTTTCGAGTCTTTGCATTGTGAACAAAAGTCAGCTTGTGTGGGAGCAAA -1400  GCCAGCTGCTCTGGGTGCAGACCCAGGAGCAGAGTGCAGAGGAGAATGAG  TCAAAGAGTTTTGTCTTCAAAAATTACATAATCGGGATTTGCTAAGAGTT -1300  TACTTTTCGGTATGGAAGACTGGAAAAGAGAAAGAAATCTTAGGTTTCTT  GAATGTTGGGTTTGGGAATAGGAAGGAAAATCGAAAACTGTAGACTTTGT -1200  CCATAAATGTTAGTGCTGGAACCCCACTCTAAAAACTTTGTTCCTTTGGA  AAACACCTCCCTTCCCCCAGAAACACACACACCCACACGAGATGGGCACG -1100  GAGTAGTCTTGAAAGACATGACAAATCACCAGACCTGGGAAGAAGCTAAA  GAGCCAGAGGGAAAAAGCCAGAAGTCGACTACCTGGGAGGAGGGATAGAC -1000  AAGAAACCAAACTAAAGGAAACTAAGGTAGGTGCTGAAAACAAGTACCAT  TTTCAACATTAACTGATGCCTTGGCTTCATGCTATAATGCCATGTTGTGT -900  TTCACTATAACCTCAGAGTGAATGAAAGAGGAAAATGGAGCTAGTTGAAA  TTTCTGCCTAAACTAGCCAGATTTTGAGACACTAAGTTATCTCAAATCAA -800  GAAATCACCCTAATGAGAATTTCAATAACCTCAGGAATTTAAGGTGCATG  CATCCCCCACCCCCCCCTTTTTTTTTTGAGACGTAGTCCCGCTCTGTTGC -700  CCAGGCTGGAGTACAGTGGCGCGATATCGGCTCACCACAACCTCTGCCTC  CCAGGTTCAAGGGATTCTCCCGCCTCAGCTTCCAGAGTAGCTGGGACTAC -600 ~ -551  AGACACCCACCACCATGCGTGGCTAATTTTTGTATTTTTAGTAGAGAGGG  GGTTTCGCCATGTTGGCCAGGCTGGTTTCAAACTCCTGACTTCAGGTGAT -500  CCGCCTGCCACGGCCTCCCAATTTACTGGGATTACAGGGGTGGGCCACCG  CGCCCGGCCTTTTTCTTAATTTTTAAAAATATTAAAGTTTTATCCCATTC -400  CTGTTGAACCATATTCCTGATTTAAAAGTTGGAAACGTGGTGAACCTAGA  AGTATTTGTTGCTGGGTTTGTCTTCAGGTTCTGTTGCTCGGTTTTCTAGT -300  TCCCCACCTAGTCTGGGTTACTCTGCAGCTACTTTTGCATTACAATGGCC  TTGGTGAGACTGGTAGACGGGATTAACTGAGAATTCACAAGGGTGGGTCA -200  GTAGGGGGTGTGCCCGCCAGGAGGGGTGGGTCTAAGGTGATAGAGCCTTC  AAATCTTTGTTAAATTTTTGGTTGGGGTGGAGAAGGAAATTAGCTGAGGA -100  CACTGCTATCTTAGAAATGCATAGAAATAGCTGAGCGTGGTGGCCTATGC  -------------------------------------------------------------------------------------- 0 |
